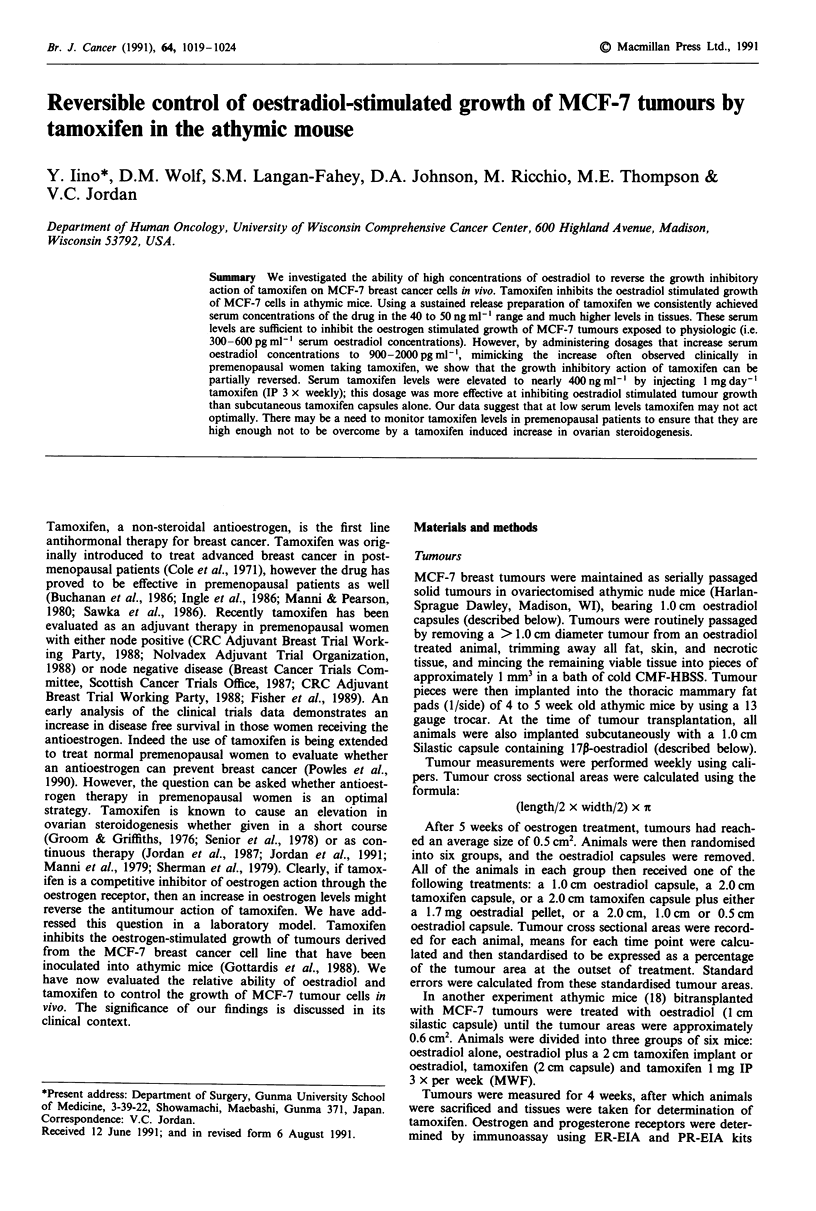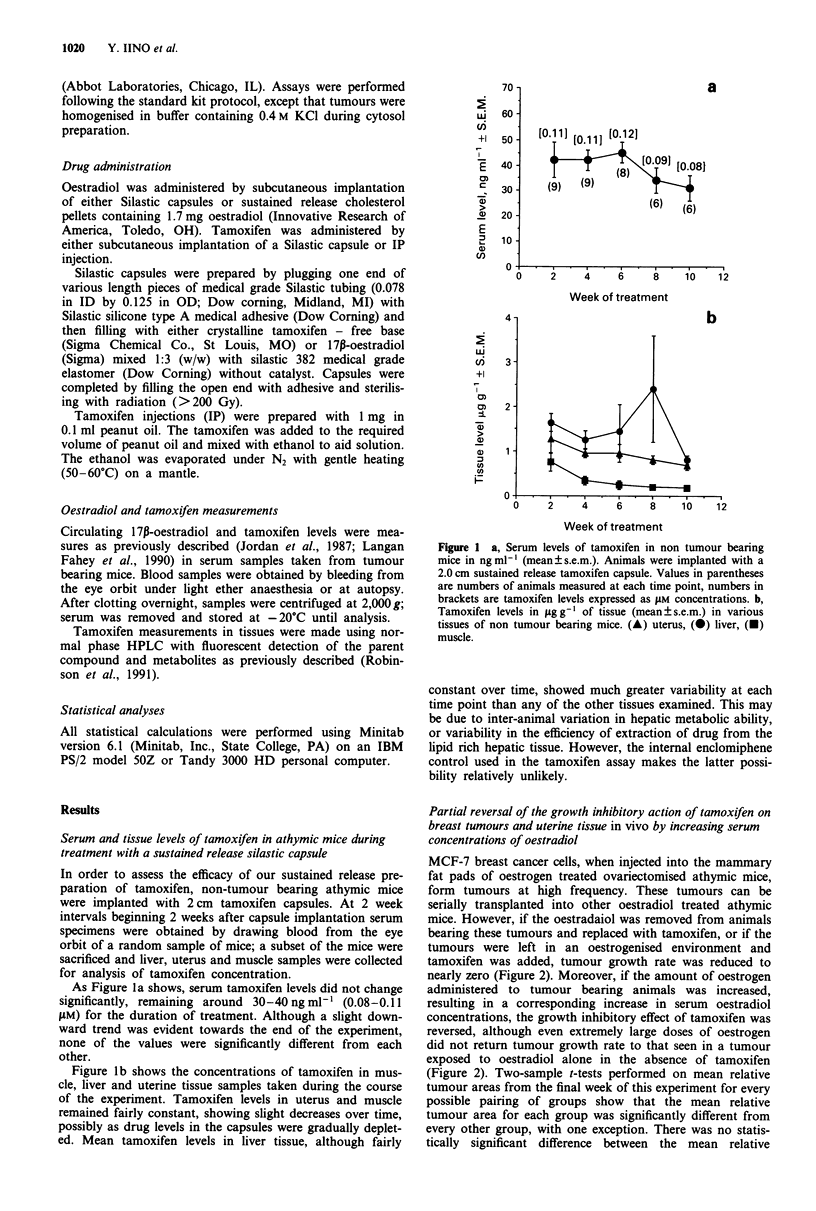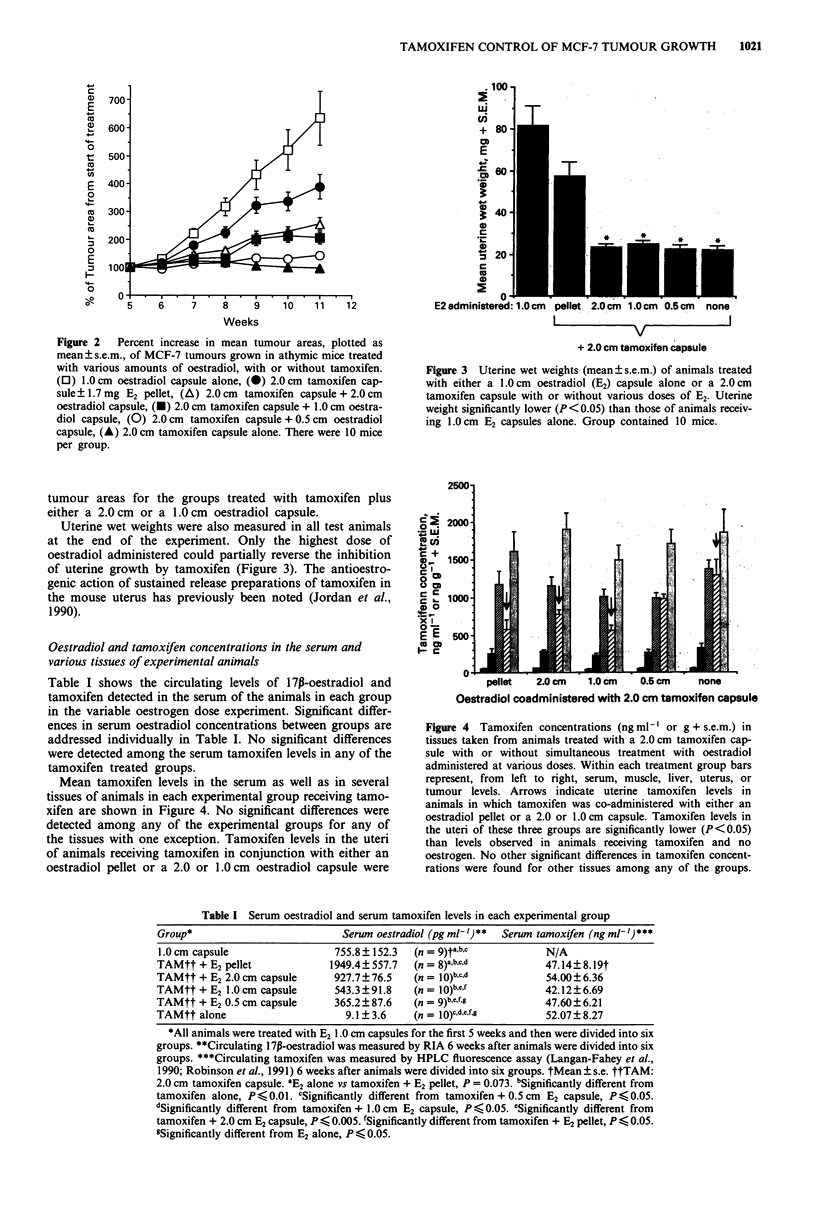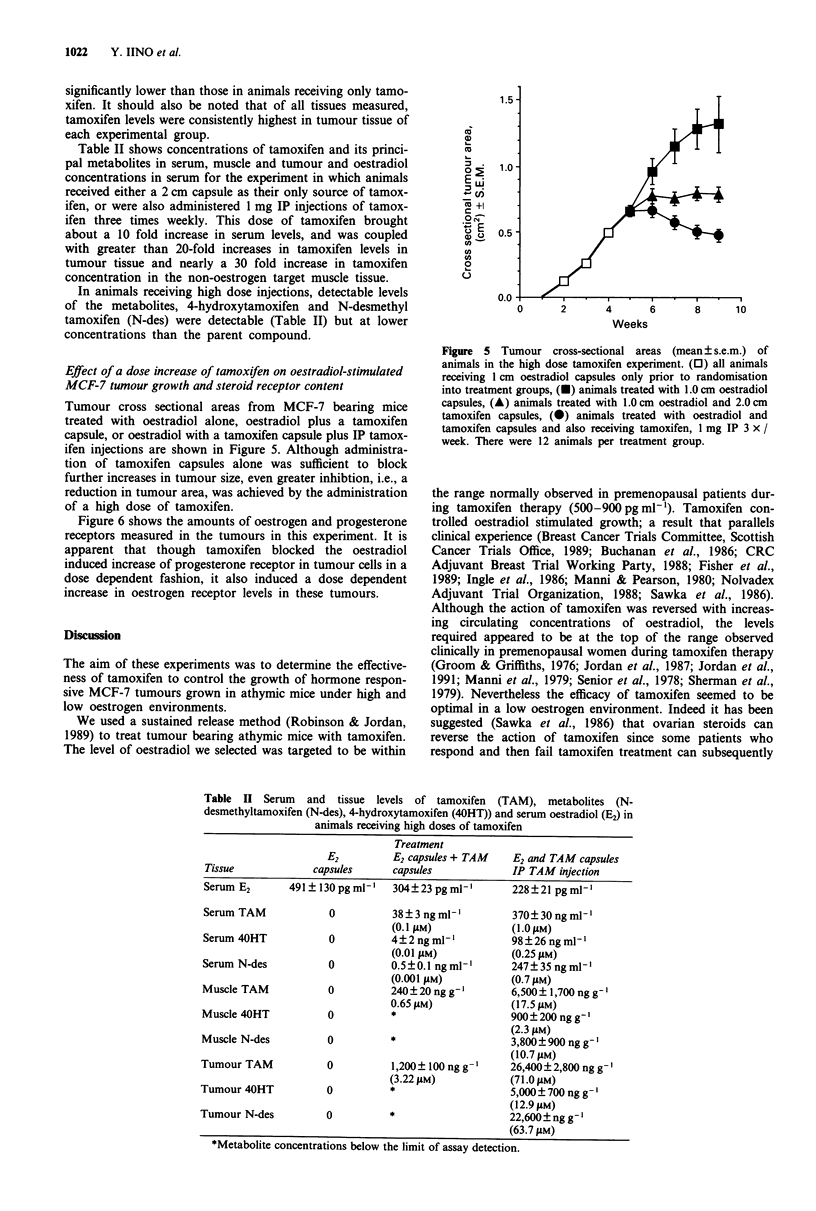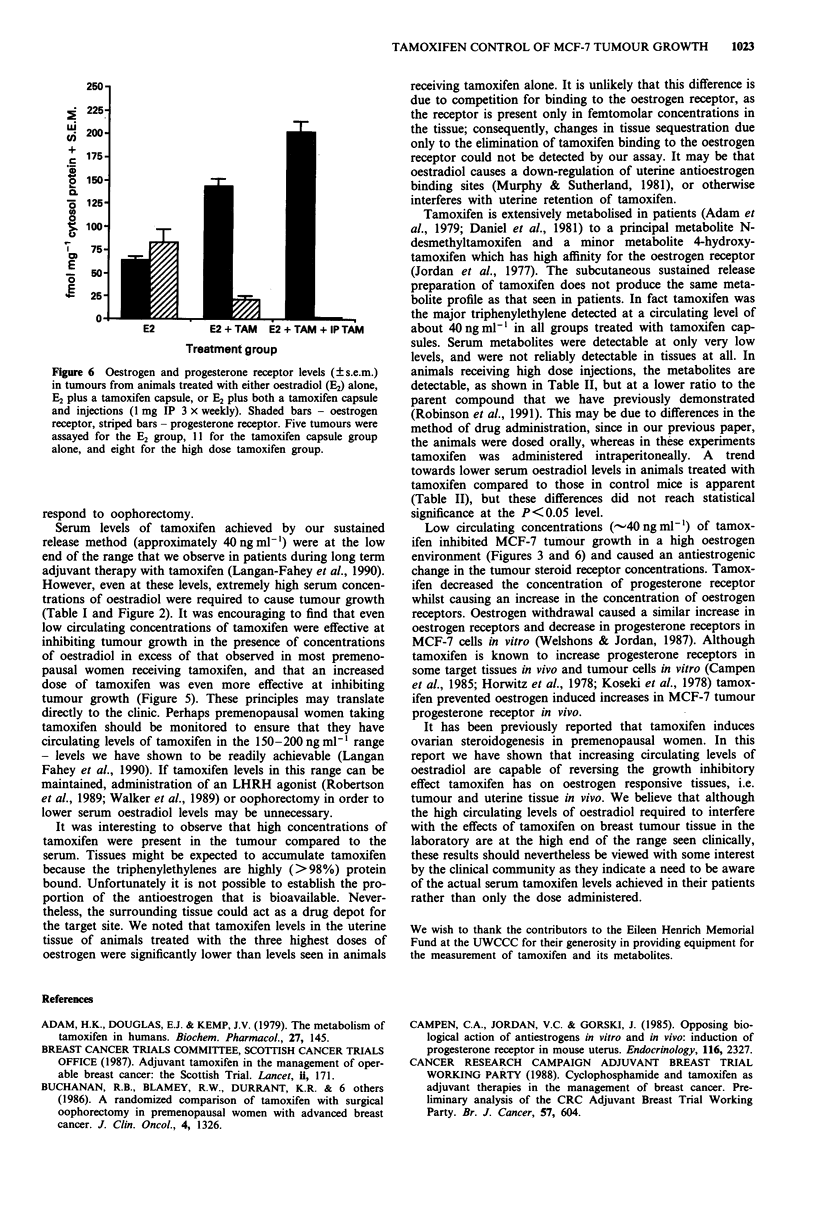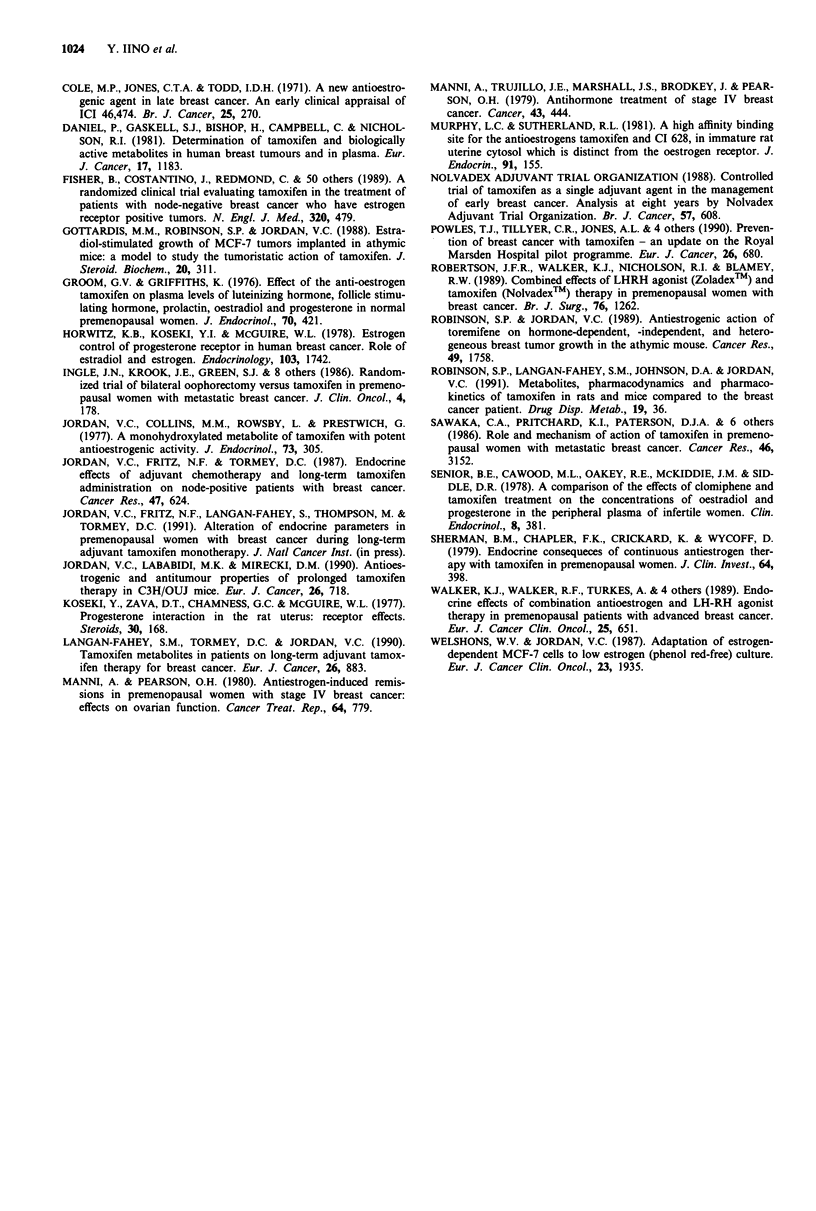# Reversible control of oestradiol-stimulated growth of MCF-7 tumours by tamoxifen in the athymic mouse.

**DOI:** 10.1038/bjc.1991.457

**Published:** 1991-12

**Authors:** Y. Iino, D. M. Wolf, S. M. Langan-Fahey, D. A. Johnson, M. Ricchio, M. E. Thompson, V. C. Jordan

**Affiliations:** Department of Human Oncology, University of Wisconsin Comprehensive Cancer Center, Madison 53792.

## Abstract

We investigated the ability of high concentrations of oestradiol to reverse the growth inhibitory action of tamoxifen on MCF-7 breast cancer cells in vivo. Tamoxifen inhibits the oestradiol stimulated growth of MCF-7 cells in athymic mice. Using a sustained release preparation of tamoxifen we consistently achieved serum concentrations of the drug in the 40 to 50 ng ml-1 range and much higher levels in tissues. These serum levels are sufficient to inhibit the oestrogen stimulated growth of MCF-7 tumours exposed to physiologic (i.e. 300-600 pg ml-1 serum oestradiol concentrations). However, by administering dosages that increase serum oestradiol concentrations to 900-2000 pg ml-1, mimicking the increase often observed clinically in premenopausal women taking tamoxifen, we show that the growth inhibitory action of tamoxifen can be partially reversed. Serum tamoxifen levels were elevated to nearly 400 ng ml-1 by injecting 1 mg day-1 tamoxifen (IP 3 x weekly); this dosage was more effective at inhibiting oestradiol stimulated tumour growth than subcutaneous tamoxifen capsules alone. Our data suggest that at low serum levels tamoxifen may not act optimally. There may be a need to monitor tamoxifen levels in premenopausal patients to ensure that they are high enough not to be overcome by a tamoxifen induced increase in ovarian steroidogenesis.


Br. J. Cancer (1991), 64, 1019-1024 © Macmillan Press Ltd., 1991

### Reversible control of oestradiol-stimulated growth of MCF-7 tumours by tamoxifen in the athymic mouse

Y. Iino*, D.M. Wolf, S.M. Langan-Fahey, D.A. Johnson, M. Ricchio, M.E. Thompson & V.C. Jordan

*Department of Human Oncology, University of Wisconsin Comprehensive Cancer Center, 600 Highland Avenue, Madison, Wisconsin 53792, USA.*

**Summary** We investigated the ability of high concentrations of oestradiol to reverse the growth inhibitory action of tamoxifen on MCF-7 breast cancer cells *in vivo*. Tamoxifen inhibits the oestradiol stimulated growth of MCF-7 cells in athymic mice. Using a sustained release preparation of tamoxifen we consistently achieved serum concentrations of the drug in the 40 to 50 ng $ml^{-1}$ range and much higher levels in tissues. These serum levels are sufficient to inhibit the oestrogen stimulated growth of MCF-7 tumours exposed to physiologic (i.e. 300–600 pg $ml^{-1}$ serum oestradiol concentrations). However, by administering dosages that increase serum oestradiol concentrations to 900–2000 pg $ml^{-1}$, mimicking the increase often observed clinically in premenopausal women taking tamoxifen, we show that the growth inhibitory action of tamoxifen can be partially reversed. Serum tamoxifen levels were elevated to nearly 400 ng $ml^{-1}$ by injecting 1 mg $day^{-1}$ tamoxifen (IP 3 × weekly); this dosage was more effective at inhibiting oestradiol stimulated tumour growth than subcutaneous tamoxifen capsules alone. Our data suggest that at low serum levels tamoxifen may not act optimally. There may be a need to monitor tamoxifen levels in premenopausal patients to ensure that they are high enough not to be overcome by a tamoxifen induced increase in ovarian steroidogenesis.

Tamoxifen, a non-steroidal antioestrogen, is the first line antihormonal therapy for breast cancer. Tamoxifen was originally introduced to treat advanced breast cancer in post-menopausal patients (Cole *et al.*, 1971), however the drug has proved to be effective in premenopausal patients as well (Buchanan *et al.*, 1986; Ingle *et al.*, 1986; Manni & Pearson, 1980; Sawka *et al.*, 1986). Recently tamoxifen has been evaluated as an adjuvant therapy in premenopausal women with either node positive (CRC Adjuvant Breast Trial Working Party, 1988; Nolvadex Adjuvant Trial Organization, 1988) or node negative disease (Breast Cancer Trials Committee, Scottish Cancer Trials Office, 1987; CRC Adjuvant Breast Trial Working Party, 1988; Fisher *et al.*, 1989). An early analysis of the clinical trials data demonstrates an increase in disease free survival in those women receiving the antioestrogen. Indeed the use of tamoxifen is being extended to treat normal premenopausal women to evaluate whether an antioestrogen can prevent breast cancer (Powles *et al.*, 1990). However, the question can be asked whether antioestrogen therapy in premenopausal women is an optimal strategy. Tamoxifen is known to cause an elevation in ovarian steroidogenesis whether given in a short course (Groom & Griffiths, 1976; Senior *et al.*, 1978) or as continuous therapy (Jordan *et al.*, 1987; Jordan *et al.*, 1991; Manni *et al.*, 1979; Sherman *et al.*, 1979). Clearly, if tamoxifen is a competitive inhibitor of oestrogen action through the oestrogen receptor, then an increase in oestrogen levels might reverse the antitumour action of tamoxifen. We have addressed this question in a laboratory model. Tamoxifen inhibits the oestrogen-stimulated growth of tumours derived from the MCF-7 breast cancer cell line that have been inoculated into athymic mice (Gottardis *et al.*, 1988). We have now evaluated the relative ability of oestradiol and tamoxifen to control the growth of MCF-7 tumour cells *in vivo*. The significance of our findings is discussed in its clinical context.

*Present address: Department of Surgery, Gunma University School of Medicine, 3-39-22, Showamachi, Maebashi, Gunma 371, Japan.
Correspondence: V.C. Jordan.
Received 12 June 1991; and in revised form 6 August 1991.

### Materials and methods

### *Tumours*

MCF-7 breast tumours were maintained as serially passaged solid tumours in ovariectomised athymic nude mice (Harlan-Sprague Dawley, Madison, WI), bearing 1.0 cm oestradiol capsules (described below). Tumours were routinely passaged by removing a $>$1.0 cm diameter tumour from an oestradiol treated animal, trimming away all fat, skin, and necrotic tissue, and mincing the remaining viable tissue into pieces of approximately 1 $mm^3$ in a bath of cold CMF-HBSS. Tumour pieces were then implanted into the thoracic mammary fat pads (1/side) of 4 to 5 week old athymic mice by using a 13 gauge trocar. At the time of tumour transplantation, all animals were also implanted subcutaneously with a 1.0 cm Silastic capsule containing 17β-oestradiol (described below).

Tumour measurements were performed weekly using calipers. Tumour cross sectional areas were calculated using the formula:

$$(\text{length}/2 \times \text{width}/2) \times \pi$$

After 5 weeks of oestrogen treatment, tumours had reached an average size of 0.5 $cm^2$. Animals were then randomised into six groups, and the oestradiol capsules were removed. All of the animals in each group then received one of the following treatments: a 1.0 cm oestradiol capsule, a 2.0 cm tamoxifen capsule, or a 2.0 cm tamoxifen capsule plus either a 1.7 mg oestradial pellet, or a 2.0 cm, 1.0 cm or 0.5 cm oestradiol capsule. Tumour cross sectional areas were recorded for each animal, means for each time point were calculated and then standardised to be expressed as a percentage of the tumour area at the outset of treatment. Standard errors were calculated from these standardised tumour areas.

In another experiment athymic mice (18) bitransplanted with MCF-7 tumours were treated with oestradiol (1 cm silastic capsule) until the tumour areas were approximately 0.6 $cm^2$. Animals were divided into three groups of six mice: oestradiol alone, oestradiol plus a 2 cm tamoxifen implant or oestradiol, tamoxifen (2 cm capsule) and tamoxifen 1 mg IP 3 × per week (MWF).

Tumours were measured for 4 weeks, after which animals were sacrificed and tissues were taken for determination of tamoxifen. Oestrogen and progesterone receptors were determined by immunoassay using ER-EIA and PR-EIA kits

(Abbot Laboratories, Chicago, IL). Assays were performed following the standard kit protocol, except that tumours were homogenised in buffer containing 0.4 M KCl during cytosol preparation.

### *Drug administration*

Oestradiol was administered by subcutaneous implantation of either Silastic capsules or sustained release cholesterol pellets containing 1.7 mg oestradiol (Innovative Research of America, Toledo, OH). Tamoxifen was administered by either subcutaneous implantation of a Silastic capsule or IP injection.

Silastic capsules were prepared by plugging one end of various length pieces of medical grade Silastic tubing (0.078 in ID by 0.125 in OD; Dow corning, Midland, MI) with Silastic silicone type A medical adhesive (Dow Corning) and then filling with either crystalline tamoxifen – free base (Sigma Chemical Co., St Louis, MO) or 17β-oestradiol (Sigma) mixed 1:3 (w/w) with silastic 382 medical grade elastomer (Dow Corning) without catalyst. Capsules were completed by filling the open end with adhesive and sterilising with radiation (>200 Gy).

Tamoxifen injections (IP) were prepared with 1 mg in 0.1 ml peanut oil. The tamoxifen was added to the required volume of peanut oil and mixed with ethanol to aid solution. The ethanol was evaporated under $N_2$ with gentle heating (50–60°C) on a mantle.

### *Oestradiol and tamoxifen measurements*

Circulating 17β-oestradiol and tamoxifen levels were measures as previously described (Jordan *et al.*, 1987; Langan Fahey *et al.*, 1990) in serum samples taken from tumour bearing mice. Blood samples were obtained by bleeding from the eye orbit under light ether anaesthesia or at autopsy. After clotting overnight, samples were centrifuged at 2,000 *g*; serum was removed and stored at −20°C until analysis.

Tamoxifen measurements in tissues were made using normal phase HPLC with fluorescent detection of the parent compound and metabolites as previously described (Robinson *et al.*, 1991).

### *Statistical analyses*

All statistical calculations were performed using Minitab version 6.1 (Minitab, Inc., State College, PA) on an IBM PS/2 model 50Z or Tandy 3000 HD personal computer.

### Results

### *Serum and tissue levels of tamoxifen in athymic mice during treatment with a sustained release silastic capsule*

In order to assess the efficacy of our sustained release preparation of tamoxifen, non-tumour bearing athymic mice were implanted with 2 cm tamoxifen capsules. At 2 week intervals beginning 2 weeks after capsule implantation serum specimens were obtained by drawing blood from the eye orbit of a random sample of mice; a subset of the mice were sacrificed and liver, uterus and muscle samples were collected for analysis of tamoxifen concentration.

As Figure 1a shows, serum tamoxifen levels did not change significantly, remaining around 30–40 ng ml$^{-1}$ (0.08–0.11 μM) for the duration of treatment. Although a slight downward trend was evident towards the end of the experiment, none of the values were significantly different from each other.

Figure 1b shows the concentrations of tamoxifen in muscle, liver and uterine tissue samples taken during the course of the experiment. Tamoxifen levels in uterus and muscle remained fairly constant, showing slight decreases over time, possibly as drug levels in the capsules were gradually depleted. Mean tamoxifen levels in liver tissue, although fairly

**Figure 1** **a**, Serum levels of tamoxifen in non tumour bearing mice in ng ml$^{-1}$ (mean ± s.e.m.). Animals were implanted with a 2.0 cm sustained release tamoxifen capsule. Values in parentheses are numbers of animals measured at each time point, numbers in brackets are tamoxifen levels expressed as μM concentrations. **b**, Tamoxifen levels in μg g$^{-1}$ of tissue (mean ± s.e.m.) in various tissues of non tumour bearing mice. (▲) uterus, (●) liver, (■) muscle.

constant over time, showed much greater variability at each time point than any of the other tissues examined. This may be due to inter-animal variation in hepatic metabolic ability, or variability in the efficiency of extraction of drug from the lipid rich hepatic tissue. However, the internal enclomiphene control used in the tamoxifen assay makes the latter possibility relatively unlikely.

### *Partial reversal of the growth inhibitory action of tamoxifen on breast tumours and uterine tissue* in vivo *by increasing serum concentrations of oestradiol*

MCF-7 breast cancer cells, when injected into the mammary fat pads of oestrogen treated ovariectomised athymic mice, form tumours at high frequency. These tumours can be serially transplanted into other oestradiol treated athymic mice. However, if the oestradaiol was removed from animals bearing these tumours and replaced with tamoxifen, or if the tumours were left in an oestrogenised environment and tamoxifen was added, tumour growth rate was reduced to nearly zero (Figure 2). Moreover, if the amount of oestrogen administered to tumour bearing animals was increased, resulting in a corresponding increase in serum oestradiol concentrations, the growth inhibitory effect of tamoxifen was reversed, although even extremely large doses of oestrogen did not return tumour growth rate to that seen in a tumour exposed to oestradiol alone in the absence of tamoxifen (Figure 2). Two-sample *t*-tests performed on mean relative tumour areas from the final week of this experiment for every possible pairing of groups show that the mean relative tumour area for each group was significantly different from every other group, with one exception. There was no statistically significant difference between the mean relative

**Figure 2** Percent increase in mean tumour areas, plotted as mean ± s.e.m., of MCF-7 tumours grown in athymic mice treated with various amounts of oestradiol, with or without tamoxifen. (□) 1.0 cm oestradiol capsule alone, (●) 2.0 cm tamoxifen capsule ± 1.7 mg $E_2$ pellet, (△) 2.0 cm tamoxifen capsule + 2.0 cm oestradiol capsule, (■) 2.0 cm tamoxifen capsule + 1.0 cm oestradiol capsule, (○) 2.0 cm tamoxifen capsule + 0.5 cm oestradiol capsule, (▲) 2.0 cm tamoxifen capsule alone. There were 10 mice per group.

**Figure 3** Uterine wet weights (mean ± s.e.m.) of animals treated with either a 1.0 cm oestradiol ($E_2$) capsule alone or a 2.0 cm tamoxifen capsule with or without various doses of $E_2$. Uterine weight significantly lower ($P<0.05$) than those of animals receiving 1.0 cm $E_2$ capsules alone. Group contained 10 mice.

tumour areas for the groups treated with tamoxifen plus either a 2.0 cm or a 1.0 cm oestradiol capsule.

Uterine wet weights were also measured in all test animals at the end of the experiment. Only the highest dose of oestradiol administered could partially reverse the inhibition of uterine growth by tamoxifen (Figure 3). The antioestrogenic action of sustained release preparations of tamoxifen in the mouse uterus has previously been noted (Jordan *et al.*, 1990).

### *Oestradiol and tamoxifen concentrations in the serum and various tissues of experimental animals*

Table I shows the circulating levels of 17β-oestradiol and tamoxifen detected in the serum of the animals in each group in the variable oestrogen dose experiment. Significant differences in serum oestradiol concentrations between groups are addressed individually in Table I. No significant differences were detected among the serum tamoxifen levels in any of the tamoxifen treated groups.

Mean tamoxifen levels in the serum as well as in several tissues of animals in each experimental group receiving tamoxifen are shown in Figure 4. No significant differences were detected among any of the experimental groups for any of the tissues with one exception. Tamoxifen levels in the uteri of animals receiving tamoxifen in conjunction with either an oestradiol pellet or a 2.0 or 1.0 cm oestradiol capsule were

**Figure 4** Tamoxifen concentrations (ng $ml^{-1}$ or g + s.e.m.) in tissues taken from animals treated with a 2.0 cm tamoxifen capsule with or without simultaneous treatment with oestradiol administered at various doses. Within each treatment group bars represent, from left to right, serum, muscle, liver, uterus, or tumour levels. Arrows indicate uterine tamoxifen levels in animals in which tamoxifen was co-administered with either an oestradiol pellet or a 2.0 or 1.0 cm capsule. Tamoxifen levels in the uteri of these three groups are significantly lower ($P<0.05$) than levels observed in animals receiving tamoxifen and no oestrogen. No other significant differences in tamoxifen concentrations were found for other tissues among any of the groups.

**Table I** Serum oestradiol and serum tamoxifen levels in each experimental group

| *Group** | *Serum oestradiol (pg $ml^{-1}$)*** | | *Serum tamoxifen (ng $ml^{-1}$)**** |
|---|---|---|---|
| 1.0 cm capsule | 755.8 ± 152.3 | ($n$ = 9)†[a,b,c] | N/A |
| TAM†† + $E_2$ pellet | 1949.4 ± 557.7 | ($n$ = 8)[a,b,c,d] | 47.14 ± 8.19† |
| TAM†† + $E_2$ 2.0 cm capsule | 927.7 ± 76.5 | ($n$ = 10)[b,c,d] | 54.00 ± 6.36 |
| TAM†† + $E_2$ 1.0 cm capsule | 543.3 ± 91.8 | ($n$ = 10)[b,e,f] | 42.12 ± 6.69 |
| TAM†† + $E_2$ 0.5 cm capsule | 365.2 ± 87.6 | ($n$ = 9)[b,e,f,g] | 47.60 ± 6.21 |
| TAM†† alone | 9.1 ± 3.6 | ($n$ = 10)[c,d,e,f,g] | 52.07 ± 8.27 |

*All animals were treated with $E_2$ 1.0 cm capsules for the first 5 weeks and then were divided into six groups. **Circulating 17β-oestradiol was measured by RIA 6 weeks after animals were divided into six groups. ***Circulating tamoxifen was measured by HPLC fluorescence assay (Langan-Fahey *et al.*, 1990; Robinson *et al.*, 1991) 6 weeks after animals were divided into six groups. †Mean ± s.e. ††TAM: 2.0 cm tamoxifen capsule. [a]$E_2$ alone *vs* tamoxifen + $E_2$ pellet, $P = 0.073$. [b]Significantly different from tamoxifen alone, $P \leqslant 0.01$. [c]Significantly different from tamoxifen + 0.5 cm $E_2$ capsule, $P \leqslant 0.05$. [d]Significantly different from tamoxifen + 1.0 cm $E_2$ capsule, $P \leqslant 0.05$. [e]Significantly different from tamoxifen + 2.0 cm $E_2$ capsule, $P \leqslant 0.005$. [f]Significantly different from tamoxifen + $E_2$ pellet, $P \leqslant 0.05$. [g]Significantly different from $E_2$ alone, $P \leqslant 0.05$.

significantly lower than those in animals receiving only tamoxifen. It should also be noted that of all tissues measured, tamoxifen levels were consistently highest in tumour tissue of each experimental group.

Table II shows concentrations of tamoxifen and its principal metabolites in serum, muscle and tumour and oestradiol concentrations in serum for the experiment in which animals received either a 2 cm capsule as their only source of tamoxifen, or were also administered 1 mg IP injections of tamoxifen three times weekly. This dose of tamoxifen brought about a 10 fold increase in serum levels, and was coupled with greater than 20-fold increases in tamoxifen levels in tumour tissue and nearly a 30 fold increase in tamoxifen concentration in the non-oestrogen target muscle tissue.

In animals receiving high dose injections, detectable levels of the metabolites, 4-hydroxytamoxifen and N-desmethyl tamoxifen (N-des) were detectable (Table II) but at lower concentrations than the parent compound.

*Effect of a dose increase of tamoxifen on oestradiol-stimulated MCF-7 tumour growth and steroid receptor content*

Tumour cross sectional areas from MCF-7 bearing mice treated with oestradiol alone, oestradiol plus a tamoxifen capsule, or oestradiol with a tamoxifen capsule plus IP tamoxifen injections are shown in Figure 5. Although administration of tamoxifen capsules alone was sufficient to block further increases in tumour size, even greater inhibtion, i.e., a reduction in tumour area, was achieved by the administration of a high dose of tamoxifen.

Figure 6 shows the amounts of oestrogen and progesterone receptors measured in the tumours in this experiment. It is apparent that though tamoxifen blocked the oestradiol induced increase of progesterone receptor in tumour cells in a dose dependent fashion, it also induced a dose dependent increase in oestrogen receptor levels in these tumours.

**Figure 5** Tumour cross-sectional areas (mean ± s.e.m.) of animals in the high dose tamoxifen experiment. (□) all animals receiving 1 cm oestradiol capsules only prior to randomisation into treatment groups, (■) animals treated with 1.0 cm oestradiol capsules, (▲) animals treated with 1.0 cm oestradiol and 2.0 cm tamoxifen capsules, (●) animals treated with oestradiol and tamoxifen capsules and also receiving tamoxifen, 1 mg IP 3 × / week. There were 12 animals per treatment group.

### Discussion

The aim of these experiments was to determine the effectiveness of tamoxifen to control the growth of hormone responsive MCF-7 tumours grown in athymic mice under high and low oestrogen environments.

We used a sustained release method (Robinson & Jordan, 1989) to treat tumour bearing athymic mice with tamoxifen. The level of oestradiol we selected was targeted to be within the range normally observed in premenopausal patients during tamoxifen therapy (500–900 pg ml$^{-1}$). Tamoxifen controlled oestradiol stimulated growth; a result that parallels clinical experience (Breast Cancer Trials Committee, Scottish Cancer Trials Office, 1989; Buchanan *et al.*, 1986; CRC Adjuvant Breast Trial Working Party, 1988; Fisher *et al.*, 1989; Ingle *et al.*, 1986; Manni & Pearson, 1980; Nolvadex Adjuvant Trial Organization, 1988; Sawka *et al.*, 1986). Although the action of tamoxifen was reversed with increasing circulating concentrations of oestradiol, the levels required appeared to be at the top of the range observed clinically in premenopausal women during tamoxifen therapy (Groom & Griffiths, 1976; Jordan *et al.*, 1987; Jordan *et al.*, 1991; Manni *et al.*, 1979; Senior *et al.*, 1978; Sherman *et al.*, 1979). Nevertheless the efficacy of tamoxifen seemed to be optimal in a low oestrogen environment. Indeed it has been suggested (Sawka *et al.*, 1986) that ovarian steroids can reverse the action of tamoxifen since some patients who respond and then fail tamoxifen treatment can subsequently

**Table II** Serum and tissue levels of tamoxifen (TAM), metabolites (N-desmethyltamoxifen (N-des), 4-hydroxytamoxifen (40HT)) and serum oestradiol ($E_2$) in animals receiving high doses of tamoxifen

| *Tissue* | *$E_2$ capsules* | *Treatment* *$E_2$ capsules + TAM capsules* | *$E_2$ and TAM capsules IP TAM injection* |
|---|---|---|---|
| Serum $E_2$ | 491 ± 130 pg ml$^{-1}$ | 304 ± 23 pg ml$^{-1}$ | 228 ± 21 pg ml$^{-1}$ |
| Serum TAM | 0 | 38 ± 3 ng ml$^{-1}$ (0.1 μM) | 370 ± 30 ng ml$^{-1}$ (1.0 μM) |
| Serum 40HT | 0 | 4 ± 2 ng ml$^{-1}$ (0.01 μM) | 98 ± 26 ng ml$^{-1}$ (0.25 μM) |
| Serum N-des | 0 | 0.5 ± 0.1 ng ml$^{-1}$ (0.001 μM) | 247 ± 35 ng ml$^{-1}$ (0.7 μM) |
| Muscle TAM | 0 | 240 ± 20 ng g$^{-1}$ 0.65 μM) | 6,500 ± 1,700 ng g$^{-1}$ (17.5 μM) |
| Muscle 40HT | 0 | * | 900 ± 200 ng g$^{-1}$ (2.3 μM) |
| Muscle N-des | 0 | * | 3,800 ± 900 ng g$^{-1}$ (10.7 μM) |
| Tumour TAM | 0 | 1,200 ± 100 ng g$^{-1}$ (3.22 μM) | 26,400 ± 2,800 ng g$^{-1}$ (71.0 μM) |
| Tumour 40HT | 0 | * | 5,000 ± 700 ng g$^{-1}$ (12.9 μM) |
| Tumour N-des | 0 | * | 22,600 ± ng g$^{-1}$ (63.7 μM) |

*Metabolite concentrations below the limit of assay detection.

**Figure 6** Oestrogen and progesterone receptor levels (±s.e.m.) in tumours from animals treated with either oestradiol ($E_2$) alone, $E_2$ plus a tamoxifen capsule, or $E_2$ plus both a tamoxifen capsule and injections (1 mg IP 3 × weekly). Shaded bars – oestrogen receptor, striped bars – progesterone receptor. Five tumours were assayed for the $E_2$ group, 11 for the tamoxifen capsule group alone, and eight for the high dose tamoxifen group.

respond to oophorectomy.

Serum levels of tamoxifen achieved by our sustained release method (approximately 40 ng ml$^{-1}$) were at the low end of the range that we observe in patients during long term adjuvant therapy with tamoxifen (Langan-Fahey *et al.*, 1990). However, even at these levels, extremely high serum concentrations of oestradiol were required to cause tumour growth (Table I and Figure 2). It was encouraging to find that even low circulating concentrations of tamoxifen were effective at inhibiting tumour growth in the presence of concentrations of oestradiol in excess of that observed in most premenopausal women receiving tamoxifen, and that an increased dose of tamoxifen was even more effective at inhibiting tumour growth (Figure 5). These principles may translate directly to the clinic. Perhaps premenopausal women taking tamoxifen should be monitored to ensure that they have circulating levels of tamoxifen in the 150–200 ng ml$^{-1}$ range – levels we have shown to be readily achievable (Langan Fahey *et al.*, 1990). If tamoxifen levels in this range can be maintained, administration of an LHRH agonist (Robertson *et al.*, 1989; Walker *et al.*, 1989) or oophorectomy in order to lower serum oestradiol levels may be unnecessary.

It was interesting to observe that high concentrations of tamoxifen were present in the tumour compared to the serum. Tissues might be expected to accumulate tamoxifen because the triphenylethylenes are highly (>98%) protein bound. Unfortunately it is not possible to establish the proportion of the antioestrogen that is bioavailable. Nevertheless, the surrounding tissue could act as a drug depot for the target site. We noted that tamoxifen levels in the uterine tissue of animals treated with the three highest doses of oestrogen were significantly lower than levels seen in animals receiving tamoxifen alone. It is unlikely that this difference is due to competition for binding to the oestrogen receptor, as the receptor is present only in femtomolar concentrations in the tissue; consequently, changes in tissue sequestration due only to the elimination of tamoxifen binding to the oestrogen receptor could not be detected by our assay. It may be that oestradiol causes a down-regulation of uterine antioestrogen binding sites (Murphy & Sutherland, 1981), or otherwise interferes with uterine retention of tamoxifen.

Tamoxifen is extensively metabolised in patients (Adam *et al.*, 1979; Daniel *et al.*, 1981) to a principal metabolite N-desmethyltamoxifen and a minor metabolite 4-hydroxytamoxifen which has high affinity for the oestrogen receptor (Jordan *et al.*, 1977). The subcutaneous sustained release preparation of tamoxifen does not produce the same metabolite profile as that seen in patients. In fact tamoxifen was the major triphenylethylene detected at a circulating level of about 40 ng ml$^{-1}$ in all groups treated with tamoxifen capsules. Serum metabolites were detectable at only very low levels, and were not reliably detectable in tissues at all. In animals receiving high dose injections, the metabolites are detectable, as shown in Table II, but at a lower ratio to the parent compound that we have previously demonstrated (Robinson *et al.*, 1991). This may be due to differences in the method of drug administration, since in our previous paper, the animals were dosed orally, whereas in these experiments tamoxifen was administered intraperitoneally. A trend towards lower serum oestradiol levels in animals treated with tamoxifen compared to those in control mice is apparent (Table II), but these differences did not reach statistical significance at the $P<0.05$ level.

Low circulating concentrations (~40 ng ml$^{-1}$) of tamoxifen inhibited MCF-7 tumour growth in a high oestrogen environment (Figures 3 and 6) and caused an antiestrogenic change in the tumour steroid receptor concentrations. Tamoxifen decreased the concentration of progesterone receptor whilst causing an increase in the concentration of oestrogen receptors. Oestrogen withdrawal caused a similar increase in oestrogen receptors and decrease in progesterone receptors in MCF-7 cells *in vitro* (Welshons & Jordan, 1987). Although tamoxifen is known to increase progesterone receptors in some target tissues *in vivo* and tumour cells *in vitro* (Campen *et al.*, 1985; Horwitz *et al.*, 1978; Koseki *et al.*, 1978) tamoxifen prevented oestrogen induced increases in MCF-7 tumour progesterone receptor *in vivo*.

It has been previously reported that tamoxifen induces ovarian steroidogenesis in premenopausal women. In this report we have shown that increasing circulating levels of oestradiol are capable of reversing the growth inhibitory effect tamoxifen has on oestrogen responsive tissues, i.e. tumour and uterine tissue *in vivo*. We believe that although the high circulating levels of oestradiol required to interfere with the effects of tamoxifen on breast tumour tissue in the laboratory are at the high end of the range seen clinically, these results should nevertheless be viewed with some interest by the clinical community as they indicate a need to be aware of the actual serum tamoxifen levels achieved in their patients rather than only the dose administered.

We wish to thank the contributors to the Eileen Henrich Memorial Fund at the UWCCC for their generosity in providing equipment for the measurement of tamoxifen and its metabolites.

### References

ADAM, H.K., DOUGLAS, E.J. & KEMP, J.V. (1979). The metabolism of tamoxifen in humans. *Biochem. Pharmacol.*, **27**, 145.

BREAST CANCER TRIALS COMMITTEE, SCOTTISH CANCER TRIALS OFFICE (1987). Adjuvant tamoxifen in the management of operable breast cancer: the Scottish Trial. *Lancet*, **ii**, 171.

BUCHANAN, R.B., BLAMEY, R.W., DURRANT, K.R. & 6 others (1986). A randomized comparison of tamoxifen with surgical oophorectomy in premenopausal women with advanced breast cancer. *J. Clin. Oncol.*, **4**, 1326.

CAMPEN, C.A., JORDAN, V.C. & GORSKI, J. (1985). Opposing biological action of antiestrogens *in vitro* and *in vivo*: induction of progesterone receptor in mouse uterus. *Endocrinology*, **116**, 2327.

CANCER RESEARCH CAMPAIGN ADJUVANT BREAST TRIAL WORKING PARTY (1988). Cyclophosphamide and tamoxifen as adjuvant therapies in the management of breast cancer. Preliminary analysis of the CRC Adjuvant Breast Trial Working Party. *Br. J. Cancer*, **57**, 604.

COLE, M.P., JONES, C.T.A. & TODD, I.D.H. (1971). A new antioestrogenic agent in late breast cancer. An early clinical appraisal of ICI 46,474. *Br. J. Cancer*, **25**, 270.

DANIEL, P., GASKELL, S.J., BISHOP, H., CAMPBELL, C. & NICHOLSON, R.I. (1981). Determination of tamoxifen and biologically active metabolites in human breast tumours and in plasma. *Eur. J. Cancer*, **17**, 1183.

FISHER, B., COSTANTINO, J., REDMOND, C. & 50 others (1989). A randomized clinical trial evaluating tamoxifen in the treatment of patients with node-negative breast cancer who have estrogen receptor positive tumors. *N. Engl. J. Med.*, **320**, 479.

GOTTARDIS, M.M., ROBINSON, S.P. & JORDAN, V.C. (1988). Estradiol-stimulated growth of MCF-7 tumors implanted in athymic mice: a model to study the tumoristatic action of tamoxifen. *J. Steroid. Biochem.*, **20**, 311.

GROOM, G.V. & GRIFFITHS, K. (1976). Effect of the anti-oestrogen tamoxifen on plasma levels of luteinizing hormone, follicle stimulating hormone, prolactin, oestradiol and progesterone in normal premenopausal women. *J. Endocrinol.*, **70**, 421.

HORWITZ, K.B., KOSEKI, Y.I. & MCGUIRE, W.L. (1978). Estrogen control of progesterone receptor in human breast cancer. Role of estradiol and estrogen. *Endocrinology*, **103**, 1742.

INGLE, J.N., KROOK, J.E., GREEN, S.J. & 8 others (1986). Randomized trial of bilateral oophorectomy versus tamoxifen in premenopausal women with metastatic breast cancer. *J. Clin. Oncol.*, **4**, 178.

JORDAN, V.C., COLLINS, M.M., ROWSBY, L. & PRESTWICH, G. (1977). A monohydroxylated metabolite of tamoxifen with potent antioestrogenic activity. *J. Endocrinol.*, **73**, 305.

JORDAN, V.C., FRITZ, N.F. & TORMEY, D.C. (1987). Endocrine effects of adjuvant chemotherapy and long-term tamoxifen administration on node-positive patients with breast cancer. *Cancer Res.*, **47**, 624.

JORDAN, V.C., FRITZ, N.F., LANGAN-FAHEY, S., THOMPSON, M. & TORMEY, D.C. (1991). Alteration of endocrine parameters in premenopausal women with breast cancer during long-term adjuvant tamoxifen monotherapy. *J. Natl Cancer Inst.* (in press).

JORDAN, V.C., LABABIDI, M.K. & MIRECKI, D.M. (1990). Antioestrogenic and antitumour properties of prolonged tamoxifen therapy in C3H/OUJ mice. *Eur. J. Cancer*, **26**, 718.

KOSEKI, Y., ZAVA, D.T., CHAMNESS, G.C. & MCGUIRE, W.L. (1977). Progesterone interaction in the rat uterus: receptor effects. *Steroids*, **30**, 168.

LANGAN-FAHEY, S.M., TORMEY, D.C. & JORDAN, V.C. (1990). Tamoxifen metabolites in patients on long-term adjuvant tamoxifen therapy for breast cancer. *Eur. J. Cancer*, **26**, 883.

MANNI, A. & PEARSON, O.H. (1980). Antiestrogen-induced remissions in premenopausal women with stage IV breast cancer: effects on ovarian function. *Cancer Treat. Rep.*, **64**, 779.

MANNI, A., TRUJILLO, J.E., MARSHALL, J.S., BRODKEY, J. & PEARSON, O.H. (1979). Antihormone treatment of stage IV breast cancer. *Cancer*, **43**, 444.

MURPHY, L.C. & SUTHERLAND, R.L. (1981). A high affinity binding site for the antioestrogens tamoxifen and CI 628, in immature rat uterine cytosol which is distinct from the oestrogen receptor. *J. Endocrin.*, **91**, 155.

NOLVADEX ADJUVANT TRIAL ORGANIZATION (1988). Controlled trial of tamoxifen as a single adjuvant agent in the management of early breast cancer. Analysis at eight years by Nolvadex Adjuvant Trial Organization. *Br. J. Cancer*, **57**, 608.

POWLES, T.J., TILLYER, C.R., JONES, A.L. & 4 others (1990). Prevention of breast cancer with tamoxifen – an update on the Royal Marsden Hospital pilot programme. *Eur. J. Cancer*, **26**, 680.

ROBERTSON, J.F.R., WALKER, K.J., NICHOLSON, R.I. & BLAMEY, R.W. (1989). Combined effects of LHRH agonist (Zoladex$^{TM}$) and tamoxifen (Nolvadex$^{TM}$) therapy in premenopausal women with breast cancer. *Br. J. Surg.*, **76**, 1262.

ROBINSON, S.P. & JORDAN, V.C. (1989). Antiestrogenic action of toremifene on hormone-dependent, -independent, and heterogeneous breast tumor growth in the athymic mouse. *Cancer Res.*, **49**, 1758.

ROBINSON, S.P., LANGAN-FAHEY, S.M., JOHNSON, D.A. & JORDAN, V.C. (1991). Metabolites, pharmacodynamics and pharmacokinetics of tamoxifen in rats and mice compared to the breast cancer patient. *Drug Disp. Metab.*, **19**, 36.

SAWAKA, C.A., PRITCHARD, K.I., PATERSON, D.J.A. & 6 others (1986). Role and mechanism of action of tamoxifen in premenopausal women with metastatic breast cancer. *Cancer Res.*, **46**, 3152.

SENIOR, B.E., CAWOOD, M.L., OAKEY, R.E., MCKIDDIE, J.M. & SIDDLE, D.R. (1978). A comparison of the effects of clomiphene and tamoxifen treatment on the concentrations of oestradiol and progesterone in the peripheral plasma of infertile women. *Clin. Endocrinol.*, **8**, 381.

SHERMAN, B.M., CHAPLER, F.K., CRICKARD, K. & WYCOFF, D. (1979). Endocrine conseqeuces of continuous antiestrogen therapy with tamoxifen in premenopausal women. *J. Clin. Invest.*, **64**, 398.

WALKER, K.J., WALKER, R.F., TURKES, A. & 4 others (1989). Endocrine effects of combination antioestrogen and LH-RH agonist therapy in premenopausal patients with advanced breast cancer. *Eur. J. Cancer Clin. Oncol.*, **25**, 651.

WELSHONS, W.V. & JORDAN, V.C. (1987). Adaptation of estrogen-dependent MCF-7 cells to low estrogen (phenol red-free) culture. *Eur. J. Cancer Clin. Oncol.*, **23**, 1935.